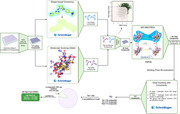# Identification of Chemical Tool Compounds to Investigate the Role of Lyn Kinase in TREM2‐Mediated Microglia Activation and Phagocytosis

**DOI:** 10.1002/alz.086617

**Published:** 2025-01-09

**Authors:** Pathum M Weerawarna, Michael T Robo, Shaoyou Chu, Emily R Mason, Chris Davis, Steven P Angus, Timothy I. Richardson

**Affiliations:** ^1^ Indiana University School of Medicine, Indianapolis, IN USA; ^2^ Indiana Bioscience Research Institute, Indianapolis, IN USA

## Abstract

**Background:**

Lyn kinase, a member of the Src family of tyrosine kinases, predominantly phosphorylates ITIM and ITAM motifs linked to immune receptors and adaptor proteins, and is emerging as a target for Alzheimer’s disease (AD). The role of Lyn in TREM2‐mediated microglial activation and phagocytosis, a critical pathway for clearing Aβ plaques, remains unclear and potent, selective, and brain penetrant Lyn inhibitors are unavailable. In this study, we report the characterization of Lyn kinase inhibitors from the literature as well as the establishment of an advanced virtual screening platform at the IUSM‐Purdue‐TREAT‐AD center to identify new type II Lyn inhibitors suitable as molecular probes.

**Method:**

We first performed a thorough literature survey and found 14 reported Lyn kinase inhibitors. We then validated their Lyn inhibitor activities and Lyn selectivities using the HotSpot kinase assay. We tested these compounds for microglia activation in a high‐content imaging assay using HMC3 (human) and BV2 (mouse) microglia‐like cell lines. We also performed kinome profiling in these cells to evaluate cellular target engagement and selectivity. Finally, we screened a million‐compounds using a computational pipeline that combined molecular docking, shape‐based screening, and MD simulations to identify novel and potent type II Lyn kinase inhibitors.

**Result:**

Our findings revealed that Type I inhibitors, particularly Saracatinib and Bosutinib, potently inhibit Lyn within the picomolar (pM) range. On the other hand, Type II inhibitors, such as Masitinib and Imatinib, displayed pronounced >20‐fold selectivity for Lyn over Hck with low nM Lyn inhibitor activities. Saracatinib and Bosutinib significantly induced phagocytosis in HMC3 cells, whereas Type II inhibitors demonstrated moderate activity in both HMC3 and BV2 cells. Our virtual screening platform identified a new type II Lyn inhibitor with picomolar activity and good Lyn/Hck selectivity.

**Conclusion:**

We have successfully evaluated previously reported inhibitors and introduced a novel type II Lyn kinase inhibitor with picomolar (pM) activities suitable for use as chemical probes to investigate the role of Lyn in TREM2‐mediated microglial activation.